# Structure of the N-terminal domain of ClpC1 in complex with the antituberculosis natural product ecumicin reveals unique binding interactions

**DOI:** 10.1107/S2059798320004027

**Published:** 2020-04-23

**Authors:** Nina M. Wolf, Hyun Lee, Daniel Zagal, Joo-Won Nam, Dong-Chan Oh, Hanki Lee, Joo-Won Suh, Guido F. Pauli, Sanghyun Cho, Celerino Abad-Zapatero

**Affiliations:** a Institute for Tuberculosis Research, College of Pharmacy, University of Illinois at Chicago, Chicago, IL 60612, USA; b Biophysics Core at the Research Resource Center, University of Illinois at Chicago, Chicago, IL 60612, USA; c Department of Pharmaceutical Sciences, College of Pharmacy, University of Illinois at Chicago, Chicago, IL 60612, USA; d Center for Biomolecular Sciences, College of Pharmacy, University of Illinois at Chicago, Chicago, IL 60612, USA; e College of Pharmacy, Yeungnam University, Gyeongsan-si, Gyeongsangbuk-do 38541, Republic of Korea; f Natural Products Research Institute, College of Pharmacy, Seoul National University, Seoul 08826, Republic of Korea; g Center for Nutraceutical and Pharmaceutical Materials, Myongji University, Cheoin-gu, Yongin-si, Gyeonggi-do 17058, Republic of Korea

**Keywords:** ClpC1, ecumicin, AAA+ ATPases, ohmyungsamicins, *Mycobacterium tuberculosis*

## Abstract

The biological processes related to protein homeostasis in *Mycobacterium tuberculosis*, the etiologic agent of tuberculosis, have recently been established as critical pathways for therapeutic intervention. Proteins of particular interest are ClpC1 and the ClpC1–ClpP1–ClpP2 proteasome complex. The structure of the potent antituberculosis macrocyclic depsipeptide ecumicin complexed with the N-terminal domain of ClpC1 (ClpC1-NTD) is presented here. Crystals of the ClpC1-NTD–ecumicin complex were monoclinic (unit-cell parameters *a* = 80.0, *b* = 130.0, *c* = 112.0 Å, β = 90.07°; space group *P*2_1_; 12 complexes per asymmetric unit) and diffracted to 2.5 Å resolution. The structure was solved by molecular replacement using the self-rotation function to resolve space-group ambiguities. The new structure of the ecumicin complex showed a unique 1:2 (target:ligand) stoichiometry exploiting the intramolecular dyad in the α-helical fold of the target N-terminal domain. The structure of the ecumicin complex unveiled extensive interactions in the uniquely extended N-terminus, a critical binding site for the known cyclopeptide complexes. This structure, in comparison with the previously reported rufomycin I complex, revealed unique features that could be relevant for understanding the mechanism of action of these potential antituberculosis drug leads. Comparison of the ecumicin complex and the ClpC1-NTD-L92S/L96P double-mutant structure with the available structures of rufomycin I and cyclomarin A complexes revealed a range of conformational changes available to this small N-terminal helical domain and the minor helical alterations involved in the antibiotic-resistance mechanism. The different modes of binding and structural alterations could be related to distinct modes of action.

## Abstract 2

Comparison of the structures of ClpC1–ecumicin and ClpC1–rufomycin complexes reveals unique interactions that are relevant to their modes of action.

## 1. Introduction   

With 1.5 million deaths in 2018, tuberculosis (TB) remains one of the top ten causes of death from a single infectious agent (World Health Organization, 2018 ▸). While current treatments for tuberculosis infection can cure the disease in many cases, the regimen is tedious and often inefficient. In addition, the need for a novel treatment is heightened by the increasing numbers of multidrug-resistant, extensively drug-resistant and totally drug-resistant strains of TB. The emergence of drug resistance in *Mycobacterium tuberculosis* (*Mtb*) has led to a number of natural product search campaigns (Baptista *et al.*, 2018 ▸; Quan *et al.*, 2017 ▸), with some promising successes such as the acyldepsipeptides (Famulla *et al.*, 2016 ▸).

Proper protein homeostasis is a vital aspect of bacterial growth and, in some cases, of bacterial virulence (Lupoli *et al.*, 2018 ▸; Culp & Wright, 2017 ▸). Of the bacterial proteasome complexes, Clp is the most validated therapeutic target (Raju *et al.*, 2012 ▸; Lee & Suh, 2016 ▸; Zhou *et al.*, 2020 ▸). The essential ClpC1–ClpP1–ClpP2 proteasome complex of *Mtb* has recently become the focus of several drug-discovery campaigns (Raju *et al.*, 2012 ▸; Lee & Suh, 2016 ▸). ClpC1 is an ATP-dependent homohexamer that is responsible for substrate recognition and unfolding. The proteasome, which is composed of a homohexamer of ClpC1, a heptamer of ClpP1 and a heptamer of ClpP2, must come together to form a functional complex. Through binding to ClpP2, acyldepsipeptides (ADEPs) have been shown to decouple the association of ClpC1 with ClpP1–ClpP2, open the pore of the complex and cause uncontrolled proteolysis (Famulla *et al.*, 2016 ▸).

Several macrocyclic peptides that specifically interact with ClpC1 have been identified to disrupt the normal functioning of the proteasome (Lupoli *et al.*, 2018 ▸). Cyclomarin A (CYMA) is structurally similar to the heptapeptide rufomycin I (RUF-I), while ohmyungsamycin A (OMS-A) and ohmyungsamycin B (OMS-B) are structurally similar to ecumicin (ECU) (Um *et al.* 2013 ▸). ECU is a tridecapeptide containing ten amino acids in a depsipeptide cycle with an extended tail of three amino acids. ECU and lassomycin (Gavrish *et al.*, 2014 ▸), a third type of cyclopeptide, both decrease the proteolytic activity of the ClpC1–ClpP1–ClpP2 complex and activate the ATPase activity (Choules *et al.*, 2019 ▸; Gavrish *et al.*, 2014 ▸). CYMA and RUF-I have opposite effects on proteolysis, with activation by CYMA and inhibition by RUF-I (Schmitt *et al.*, 2011 ▸; Choules *et al.*, 2019 ▸). CYMA has been proposed to have no effect on the ATPase activity (Schmitt *et al.*, 2011 ▸), while RUF-I has been observed to have no significant effect (Choules *et al.*, 2019 ▸).

ClpC1 is a 95 kDa caseinolytic AAA+ protein with an N-terminal domain (NTD) and two nucleotide-binding domains (D1 and D2). The NTD (amino acids 1–145) has been studied extensively to determine the most critical residues for the binding of ClpC1 to several natural cyclopeptides with potent anti-TB activity. The Protein Data Bank (PDB) contains several relevant X-ray structures, including those of apo *Mtb*ClpC1-NTD (PDB entry 3wdb), *Mtb*ClpC1-NTD complexed with CYMA (PDB entry 3wdc) and F2Y (PDB entry 3wdd) and F80Y (PDB entry 3wde) mutants of *Mtb*ClpC1-NTD (Vasudevan *et al.*, 2013 ▸). In addition, the structure of *Mtb*ClpC1-NTD complexed with a related cyclic heptapeptide, RUF-I (PDB entry 6cn8), has recently been reported (Wolf *et al.*, 2019 ▸). Structures of the complex of full-length ClpC from *Bacillus subtilis* with MecA, another regulatory element of proteostasis (PDB entry 3j3s; Liu *et al.*, 2013 ▸), of *B. subtilis* ClpC-NTD-D1–MecA (PDB entry 3pxg; Liu *et al.*, 2013 ▸) and of *Mtb*ClpP1P2 (PDB entry 6iw7; R. Bao, Y. Z., Luo, Y. B. Zhu, Y. Yang and Y. Z. Zhou, unpublished work) are also available. The complexes with the structurally related CYMA and RUF-I showed the same binding mode and site, indicating that a specific site at the NTD–D1 interface is essential for enzymatic function. However, the roles and the modes of action of the various potent cyclopeptides that drastically affect protein homeostasis are still to be understood.

Owing to the structural and functional differences in the cyclopeptides, their binding to ClpC1 may vary greatly and disrupt normal regulation using a variety of mechanisms. The present study sought to answer these questions by determining their three-dimensional binding and to extract any possible understanding of their mechanism of action on ClpC1 as well as their interactions with the proteolytic component (ClpP1–ClpP2). Initial factors important for analysis of the structure–activity relationship of some macrocyclic analogs are also presented.

## 2. Materials and methods   

### 2.1. Protein expression, purification and crystallization   

The preparation of ClpC1-NTD (residues 1–145 plus a C-terminal His tag) was as described previously (Wolf *et al.*, 2019 ▸). To obtain the complex with ECU, purified ClpC1-NTD samples were incubated with ECU overnight in an approximately 1:1.2 molar ratio (ClpC1-NTD:ECU). The protein complex was mixed in a 1:1 ratio with conditions from several crystallization screening kits (MCSG1–4 and The JSCG Core Suites I–IV) and crystals were grown at 16–19°C using a Tecan Freedom EVO 200 robot at the University of Illinois at Chicago Research Resource Center.

### 2.2. Data collection and processing   

Single crystals were cooled in liquid nitrogen and diffraction data were collected on the Life Science 21-ID beamline at the Advanced Photon Source (APS), Argonne National Laboratory. The resulting crystals were initially characterized at the beamline as follows and are referred to by the codes for the corresponding PDB entries: 6pbs, orthorhombic, space group *P*222_1_, *a* = 80.06, *b* = 112.56, *c* = 130.34 Å; 6pba, space group *C*2, *a* = 44.53, *b* = 59.12, *c* = 58.79 Å, β = 97.49°; 6pbq, space group *P*2_1_, *a* = 33.60, *b* = 63.29, *c* = 36.69 Å, β = 115.41°; 6ucr, space group *P*2_1_2_1_2_1_, *a* = 38.14, *b* = 63.35, *c* = 68.30 Å. The crystals contained six, one, one and one molecule(s) of ClpC1-NTD, respectively, in the asymmetric unit (Table 1 ▸).

Data sets were collected from three different crystal forms (6pbs, 6pba and 6pbq) grown in the presence of ECU. The 6pbs crystals diffracted to approximately 2.5 Å resolution, while the 6pba and 6pbq crystals diffracted to 1.8 and 1.6 Å resolution, respectively. The experimental conditions for data collection from the three forms were as follows. For 6pbs, 480 frames of 0.5° per frame were collected with 1.25 s exposure at a crystal-to-detector distance of 370 mm on beamline 21-ID-G using a MAR300 CCD detector. The data-collection parameters for the other two crystals were similar, except for changes to the crystal-to-detector distance and the exposure time accordingly to take into account the higher resolution diffraction and different data quality (6pba, space group *C*2, 1.80 Å resolution; 6pbq, space group *P*2_1_, 1.6 Å resolution). A similar protocol was followed for data collection for the final structure 6ucr.

For 6pbs, the data were initially processed using *HKL*-2000 in the *P*2 and *P*222 classes with a suggested screw axis along the longest cell dimension (130 Å). The differences in the overall *R*_p.i.m._ values between the two data reductions at 2.5 Å resolution did not vary significantly (0.044 versus 0.041). 6pbs was subsequently reprocessed with *XDS* in two separate space groups: *P*2_1_ and *P*2_1_2_1_2_1_. For 6pba, the data were also reprocessed with *iMosflm* to 1.77 Å resolution.

### 2.3. Structure solution   

The crystal structures of the three different crystal forms (6pbs, 6pba and 6pbq) were solved by molecular replacement using the refined ClpC1-NTD structure from our earlier RUF-I complex and this proceeded without difficulty, with the corresponding asymmetric units containing six (*P*2_1_2_1_2_1_), one (*C*2) and one (*P*2_1_) molecule(s), respectively. After an initial round of refinement and examination of the electron density, none of the crystal forms appeared to contain ECU. The 6pba and 6pbq structures were further refined assuming that the higher resolution might provide a more promising avenue to the structure of the ClpC1-NTD–ECU complex. However, after extensive refinement neither of the two data sets provided convincing evidence for the presence of ECU in the crystals. The putative solution of the 6pbs crystal form in the ortho­rhombic space group could not be refined beyond an *R*_free_ of 0.45.

**Figure 1:**
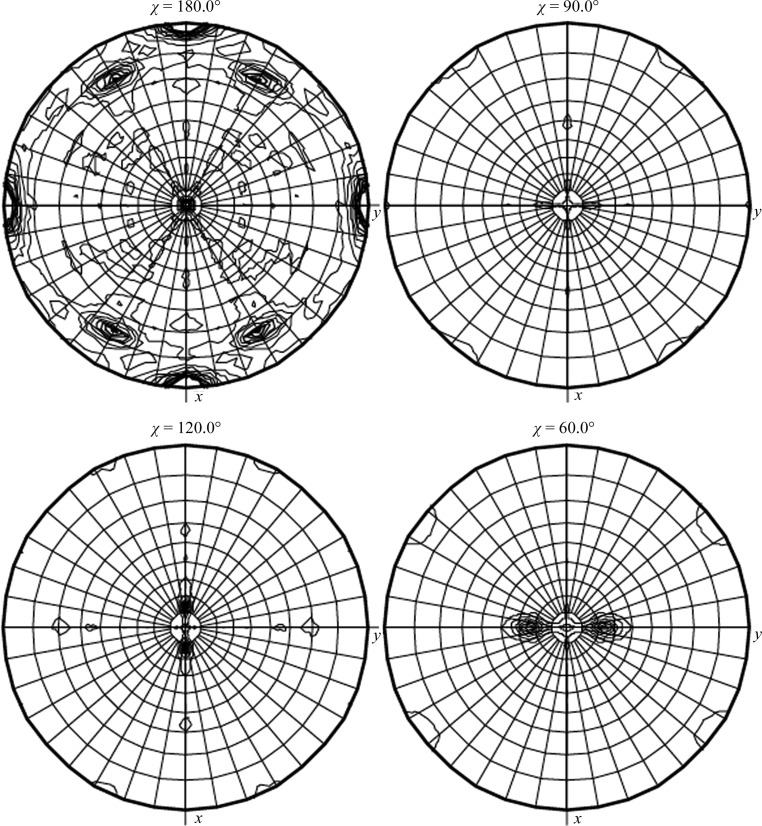
Self-rotation function of ECU complexed with ClpC1-NTD. The self-rotation function of ClpC1-NTD–ECU processed in space group *P*2_1_ contains additional orthogonal twofolds, suggesting pseudo (or nearly) orthorhombic symmetry. The presence of threefold noncrystallographic symmetry is noteworthy.

Using the extensively refined structures of 6pba and 6pbq for molecular replacement, a concerted effort was devoted to finding a satisfactory solution for the possible monoclinic 6pbs data set that contained 12 molecules in the asymmetric unit and only diffracted to 2.5 Å resolution. Surprisingly, the self-rotation function of the data set integrated and reduced as space group *P*2 provided clear evidence for non­crystallographic dimers and trimers (Fig. 1 ▸), an unexpected finding. Also, when processed in *P*2 the same data set revealed a self-rotation function that was fully consistent with pseudo-*P*222 symmetry, showing the same non­crystallographic dimers and trimers. A consistent non­crystallographic dimer was found in the search for solutions, and eventually a possible solution (*R* factor = 0.550, CC = 0.492) was found using *MOLREP* (Murshudov *et al.*, 2011 ▸) in space group *P*2_1_2_1_2_1_, with six molecules in the asymmetric unit forming a hexamer of three dimers with approximate 32 symmetry. Using this hexameric oligomer, a single solution was found using *Phaser* (McCoy *et al.*, 2007 ▸) in space group *P*2_1_2_1_2_1_ (LLG = 1140), independently supporting this hypothesis. However, initial refinement of this solution using noncrystallographic NCS restraints stalled after three cycles with the following *R* values: a starting *R*_work_ of 0.413 and *R*_free_ of 0.435 and a final *R*_work_ of 0.364 and *R*_free_ of 0.435. Releasing the NCS restraints for another three cycles ended with an *R*_work_ of 0.333 and *R*_free_ of 0.424. The electron-density maps of the partially refined structures provided strong evidence for significant changes in what appeared to be the N- and C-termini of the ClpC1-NTD molecule; in particular, the four N-terminal residues were removed in some chains to provide a ‘revised’ structure. Further partial refinement of this structure using *Phenix* (Liebschner *et al.*, 2019 ▸) reached a plateau at *R*_work_ = 0.326 and *R*_free_ = 0.418 (r.m.s.d. for bond lengths of 0.021 Å, r.m.s.d. for angles of 2.11°). Other revisions of the structure of the six ClpC1-NTD molecules failed to decrease the *R*_work_ below 0.40. The higher resolution 6pba and 6pbq structures were critical at this stage of structure solution.

The original data were then reprocessed with *XDS* (Kabsch, 2010 ▸) in both space groups *P*2_1_ and *P*2_1_2_1_2_1_. The unit-cell parameters varied in a significant way, particularly for *P*2_1_ (*a* = 80.06, *b* = 130.34, *c* = 112.56 Å, β = 90.07°). This suggested that the real space group could be monoclinic with a minor deviation of the β angle, resulting in pseudo-ortho­rhombic symmetry. Using the reprocessed data, solution of the structure was attempted by searching for two sets of six-molecule aggregates that had been partially refined before. Such a solution was immediately found (*R* factor = 0.520, CC = 0.562, TF/σ = 11.2), indicating that this was indeed the solution. The electron-density maps revealed clear density for additional chemical matter consistent with a macrocycle of the size of ECU in the proximity of all protein chains. Using the ‘LigandFit’ option in *Coot* (Emsley *et al.*, 2010 ▸), an excellent fit was found for the structure of ECU, with a conformation essentially identical to that of free ECU (Gao *et al.*, 2014 ▸). Two ECU molecules were fitted in two different ClpC1-NTD chains as a test. Using this partial solution, an initial refinement was initiated, and after three cycles the *R*_work_ decreased to 0.289 (*R*_free_ = 0.430), thus validating the solution. The full refinement was initiated from this solution containing 12 molecules of ClpC1-NTD in the asymmetric unit in space group *P*2_1_, with two ECU molecules placed in two separate chains corresponding to the best electron density for molecules in the ‘interior’ of the aggregate.

### 2.4. Refinement   

**Figure 2:**
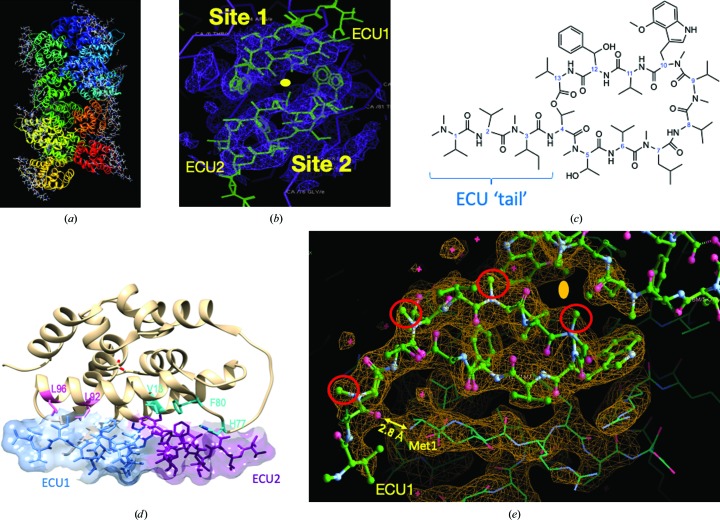
Crystallographic structure of the ClpC1-NTD–ECU complex. (*a*) Overall view of the asymmetric unit containing 12 molecules of ClpC1-NTD and 24 molecules of ECU in a 1:2 complex. (*b*) Initial 2*F*_o_ − *F*_c_ electron density for ECU showing the quality of the density (2.5 Å) and the intradomain dyad relating ECU site 1 and ECU site 2. (*c*) Two-dimensional drawing of ECU with each amino-acid carbon labeled in blue. Stereochemical details can be found in Gao *et al.* (2014 ▸). Note the depsipeptide linkage between the C-terminus of Val13 and the OH group of Thr4. (*d*) The structure of the ClpC1-NTD–ECU monomer with known resistance sites highlighted. The sites in pink confer resistance to ECU, while those in aqua confer resistance to RUF-I (Gao *et al.*, 2015 ▸; Choules *et al.*, 2019 ▸). (*e*) Refined 2*F*_o_ − *F*_c_ electron density of ECU site 1 shows unambiguous density for the entire ligand. The tail of ECU1 wraps around, forming an ‘elbow’-like pocket, and is anchored by the N-terminus of ClpC1-NTD hydrogen-bonding to the C=O of Val2 in ECU1. Four *N*-methyl groups are highlighted with red circles to emphasize the hydrophobic (upper, curved) versus hydrophilic (lower, straight) surfaces of the ECU molecule. The intradomain dyad relating the two ECU molecules is approximately vertical and in the plane of the figure in (*d*), between the two ECU molecules; the dyad is perpendicular to the plane of the image and is marked by a yellow oval in (*e*).

The full refinement of the solution found for the 6pbs crystal form in space group *P*2_1_ and with two ECU molecules was then continued by conventional methods, initially adding up to six and later 12 molecules of ECU for a full refinement. Particularly important was the complete removal of the N-terminal residues 1–4 in all chains, as significant interactions were taking place between ECU and these four residues (Met-Phe-Glu-Arg) of ClpC1-NTD, which exhibited a different orientation compared with those observed in the RUF-I complex. After the placement of six ECU molecules, the refinement parameters using NCS restraints further confirmed the correctness of the solution (*R*_work_ = 0.361, *R*_free_ = 0.367). Further restrained refinement of the six complexes (ClpC1-NTD–ECU) decreased the refinement indices to *R*_work_ = 0.337, *R*_free_ = 0.377 (r.m.s.d. for bond lengths of 0.018 Å and r.m.s.d. for angles of 1.62°). The first complete refinement of the 12 complexes in the asymmetric unit confirmed the structure (*R*_work_ = 0.271, *R*_free_ = 0.330 for a total of 7170 reflections). The complexity of the structure resulted in frequent problems with restraint descriptions as the refinement progressed, but the overall conformation of the ligand did not change significantly except for the extended ‘tail’ of the structure (Fig. 2 ▸ *c*).

The most significant result of the additional refinement was that a second ECU molecule was shown to be bound to the target, resulting in a 1:2 target:ligand stoichiometry and a 12 ClpC1-NTD:24 ECU ratio in the final structure. The partial refinement parameters along the path to the final refinement parameters (Table 1 ▸) were 12 ClpC1-NTD:24 ECU (without NCS restraints *R*_work_ = 0.246, *R*_free_ = 0.306; with NCS restraints *R*_work_ = 0.255, *R*_free_ = 0.289). Significant improvement of the stereochemistry and refinement parameters were observed when the four N-terminal residues of all of the chains were rebuilt, resulting in a rather unusual conformation for residues Met-Phe-Glu-Arg at one of the ECU sites (site 1); the second ECU molecule (site 2) has a distinct set of contacts with the ClpC1-NTD protein near the connecting loop (residues 69–80) between the two helical repeats in the ClpC1-NTD structure (Fig. 2 ▸ *b*). After solving these main issues, the refinement followed established methods.

The structure solution and refinement of the double mutant L92S/L96P of ClpC1-NTD also followed conventional protocols and revealed a more extended C-terminus than in the other three structures. Table 1 ▸ summarizes the data collection/reduction parameters as well as the final refinement statistics for the four novel structures.

### 2.5. Surface plasmon resonance (SPR) binding studies   

Recombinant DNA for full-length ClpC1 (ClpC1-FL) and ClpC1-NTD was codon-optimized, synthesized and cloned into pET-15b vector with an N-terminal His_6_-SUMO tag (GenScript, Piscataway, New Jersey, USA). ClpC1-FL mutants V14A, Q17A, K85A and L92S/L96P and the N-terminal mutants FER (residues 2–145), AAFER, MAFER, MVFER and MVAFER also had His_6_-SUMO N-terminal tags. All proteins were prepared using Ni–NTA mini spin columns (Qiagen, Germantown, Maryland, USA). The His_6_-SUMO tags were cleaved with SUMO protease prepared in-house and the cleaved proteins were separated from their tags and the SUMO protease using Ni–NTA columns. The purified proteins were then buffer-exchanged into phosphate-buffered saline with 15% glycerol using a desalting column before storage at −80°C.

SPR studies were carried out using either a Biacore T200 or a Biacore 8K as reported previously (Wolf *et al.*, 2019 ▸). In brief, ClpC1 proteins were immobilized on a CM5 sensor chip using standard amine coupling. RUF-I, ECU, OMS-A and OMS-B were isolated as described previously (Gao *et al.*, 2014 ▸; Choules *et al.*, 2019 ▸; Hur *et al.*, 2018 ▸). ECU, RUF-I and OMS-A solutions were prepared at a series of increasing concentrations and were injected onto both blank surfaces and ClpC1 protein immobilized surfaces at a flow rate of 30 µl min^−1^ at 25°C. All sensorgrams were double-referenced with blank channels and zero concentration, followed by fitting the data with two kinetic models (1:1 Langmuir and heterogeneous ligand models) using either *Biacore T200 Evaluation Software* version 3 or *Biacore 8K Insight Evaluation Software*. All experiments, excluding the N-terminal mutants, were carried out with both ClpC1-NTD and ClpC1-FL and similar results were obtained; therefore, just one set of data is provided for simplicity.

## 3. Results   

### 3.1. Structure descriptions   

#### 3.1.1. Structure of the ClpC1-NTD–ECU complex (PDB entry 6pbs)   

The asymmetric unit of the ClpC1-NTD–ECU complex (Fig. 2 ▸ *a*) contains 12 molecules of the N-terminal domain of ClpC1 (residues 1–158, including an C-terminal His tag) with two molecules of the ECU macrocycle bound to each of the target molecules in the asymmetric unit. The two bound ECU molecules are related by the pseudo-intramolecular dyad (Kar *et al.*, 2008 ▸) that relates the two α-helical domains of the N-terminal domain fold (Fig. 2 ▸ *b*). This binding mode was unexpected and presents a unique example of 1:2 (target:ligand) stoichiometry among the target complexes known for these types of natural products. The result is particularly surprising as structurally related (although smaller) hepta­cyclopeptides such as CYMA (Vasudevan *et al.*, 2013 ▸) and RUF-I (Wolf *et al.*, 2019 ▸) bound with a 1:1 ratio of target to ligand while occupying the same binding region.

The structure of the ECU ligand did not change significantly from the available structure of the isolated macrocycle (CSD 940680) and was initially fitted as a rigid body into the un­refined density. After refinement, only small deviations of the N-terminus of ClpC1 (residues 1–4) were found among the different complexes in the asymmetric unit (Supplementary Table S1).

ECU contains four *N*-methyl groups on one side of the molecule, whereas the other side is polar as the N atoms of the amide bonds are exposed (Fig. 2 ▸ *e*). In the structure of the complex the nonpolar sides of the ECU molecules face each other across the intramolecular pseudo-dyad, which provides an additional hydrophobic surface of approximately 240 Å^2^ (15% of the total) for the second ECU molecule to bind. The environment and interactions of the polar sides of the two ECU molecules, including their three-residue tails, with binding sites 1 and 2 of ClpC1-NTD are different.

In site 1, the polar side of ECU (including its three-residue tail) serves as a very effective ‘anchor and guide’ to induce a conformational change in the four N-terminal residues of ClpC1-NTD (Met1-Phe2-Glu3-Arg4) and form an extended chain that distinctively differs from the short 3_10_-helix found in the ClpC1-NTD–RUF-I complex (Wolf *et al.*, 2019 ▸). On the hydrophobic side, the two methoxy-Trp residues and the interaction between the β-hydroxy-Phe of ECU and Glu3 on the polar side of ECU are important. A distinct feature of the binding is the nonstandard β-strand conformation of Met1-Phe2-Glu3, which is facilitated by a hydrogen-bond network (Supplementary Fig. S1 and Table S2), in particular to the important carboxyl group of the Glu3 side chain (Fig. 2 ▸ *e*), and also the interaction between the N-terminal amine group of the protein and the C=O of Val2 in the tail of ECU. This conformation is probably favored owing to the presence of the *N*-methyl group of Val2 of ECU.

**Figure 3:**
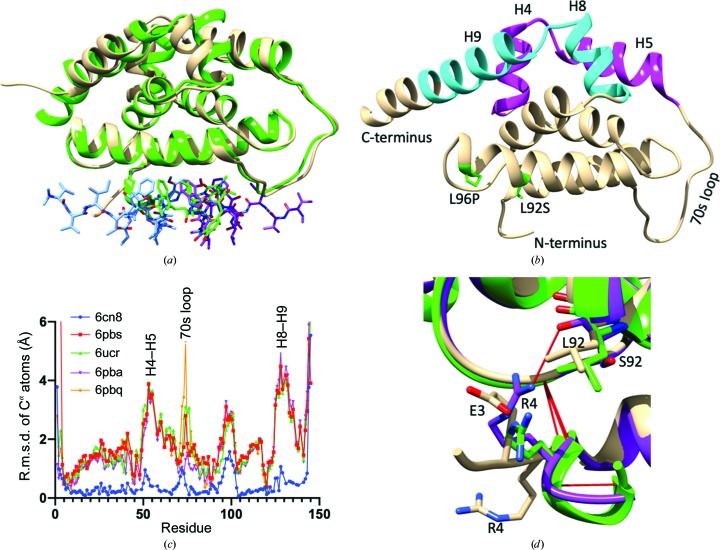
Conformational changes in ClpC1-NTD crystallographic structures. (*a*) Superposition of the complexes of ClpC1-NTD with ECU (protein, beige; ECU site 1, blue; ECU site 2, purple) and RUF-I (protein and RUF-I both shown in green). Subtle changes are seen at the peptide-binding site, with a domain movement of helices 4–5 and 8–9 caused by a twist in the 70s loop. RUF-I occupies the cyclic portion of ECU site 2. (*b*) The N-terminus of ClpC1-NTD-L92S/L96P is curved in approximately the same position as in apo ClpC1-NTD and the complexes with RUF-I and CYMA. A distortion of helix 6 is observed just before L96P. (*c*) R.m.s.d. of C^α^ atons compared with apo ClpC1-NTD (PDB entry 3wdb). There is very little movement of the backbone between the apo and RUF-I-bound forms. However, there are substantial movements of the termini, the repeat-linking 70s loop and helices 4–5 and 8–9. (*d*) Close-up view of the unusual conformation of the N-terminus of ClpC1-NTD (Met1-Phe2-Glu3-Arg4); ClpC1-NTD-L92S/L96P is in purple, ClpC1-NTD–RUF-I is in green and ClpC1-NTD–ECU is in beige. Arg4 undergoes a substantial conformational change when compared with the unbound structure in the ClpC1-NTD-L92S/L96P mutant, allowing it to hydrogen-bond (red line) from the Arg4 NH2 group to the carbonyl of Ser92. Arg4 O hydrogen-bonds to Ile103 N in the loop following helix 6 in all three structures. In addition, Arg4 N hydrogen-bonds to Met1 O in the ClpC1-NTD–RUF-I complex.

The interactions of the tail of ECU at site 2, across the intramolecular dyad, are distinct as follows. The residues corresponding to the N-terminal residues in ClpC1-NTD in the intramolecular repeat are the residues (60–78) located in the loop that links the two helical domains of the target protein. This loop is significantly more flexible in all reported structures of ClpC1-NTD: apo (PDB entry 3wdb), the CYMA complexes (PDB entries 3wdc, 3wdd and 3wde) and the RUF-I complex (PDB entry 6cn8). The key residue in this loop appears to be His77 (Fig. 3 ▸ *c*), where sizable movements are seen. Based on the quality of the electron density for the tail residues of ECU and the interacting residues in ClpC1-NTD, it is probable that ECU binds to site 1 with higher affinity than to site 2. Possibly, the binding of the ECU molecule in site 2 is facilitated by the preceding ECU binding to site 1. The occurrence of three strains of *Mtb* with resistance to ECU as a result of mutations at positions Leu92 and Leu96 in ECU binding site 1 of ClpC1 (Gao *et al.*, 2015 ▸) further supports the binding strength at this site.

The most significant differences among the 12 ClpC1-NTD molecules in the asymmetric unit of the ClpC1-NTD–ECU complex are the extent and quality of electron density for the carboxy-terminal residues. In the crystal packing, this part of the target protein interacts with the neighboring molecules, providing conformational heterogeneity. These dynamics are probably responsible for the small deviations observed from a genuine orthorhombic crystal lattice (β = 90.07°). This observation compounds with the aforementioned minor differences in the three-residue tail of ECU itself, leading to minor asymmetry between ClpC1-NTD–ECU monomers.

#### 3.1.2. Single-point mutations L92S/L96P   

Since single mutations at sites Leu92 and Leu96 of ClpC1 caused a reduced binding affinity for ECU, the crystal structure of an engineered double mutant was solved to investigate the mechanism of resistance. The mutations L92S and L92F have reduced affinity compared with ClpC1. Since the L92S mutation had a lower affinity for ClpC1-NTD, it was chosen for the double mutant. Only one mutation was found at position 96 (Wolf *et al.*, 2019 ▸). Superposition of the structures of ClpC1-NTD-L92S/L96P and the ECU complex resulted in an overall r.m.s.d. of 1.12 Å or of 0.71 Å without the three N-terminal residues (Supplementary Table S3). The N-terminus of ClpC1-NTD-L92S/L96P is a curved loop in approximately the same orientation as in the RUF-I complex and apo forms. Looking into the changes at the mutation sites, a distortion (‘kink’) in the helical backbone induced by proline and the missing nitrogen bond acceptor of the carboxyl of Ser92 was observed. This C=O bond protrudes out of the helix axis and is at an approximate hydrogen-bonding distance from NH2 of Arg4. In other structures this distance is about 5.9–7.8 Å. Accordingly, the L96P mutation apparently does not have a significant effect on the length of helix 6 as both the ClpC1-NTD-L92S/L96P and ECU complex structures terminate this helix at residue 97, while some other structures terminate at position 98. This subtle alteration is significant in relation to the binding of ECU, as the conformation of Arg4 may be important for the three previous residues of the N-terminus (Met1-Phe2-Glu3) to adopt the extended ‘ECU-binding’ conformation for site 1.

### 3.2. Structure comparisons   

#### 3.2.1. Apo structure comparisons   

Attempts to co-crystallize ECU and ClpC1-NTD initially failed, probably owing to the poor solubility of ECU. These attempts yielded two distinct high-resolution apo structures crystallized in the presence of ECU (PDB entries 6pba and 6pbq). Excluding the four N-terminal residues, the r.m.s.d.s between these two apo structures and that with bound ECU (chain *W*) are 0.43 and 0.68 Å, respectively (Supplementary Table S3), explaining the importance of these structures in solving the large asymmetric unit of the ClpC1-NTD–ECU complex. The apo structure (PDB entry 3wdb) differed significantly from the apo structures of the L96P/L92S mutant (PDB entry 6ucr), 6pba and 6pbq (r.m.s.d.s of 2.06, 2.00 and 2.07 Å, respectively; Supplementary Table S3). The largest r.m.s.d. was against the ClpC1-NTD–ECU complex (2.45 Å). This could explain the difficulty in solving the ECU complex structure by molecular replacement using PDB entry 3wdb as the search model. In addition, the N-terminus of ClpC1-NTD maintains a distinct 3_10_-helical structure in the RUF-I and CYMA complexes, rigidified by the covalent bond with Met1, while the N-terminus is more extended in the apo structures and in the ECU complex. It should be noted that the PDB entry 3wdb apo structure contains an N-terminal His tag, unlike all other structures.

Various ions from purification and the crystallization solution were found in the structures. 6pba did not contain any noteworthy ligands, but 6pbq contained a HEPES molecule from the crystallization medium, the SO_3_ group of which is in a polar pocket that is occupied by PO_4_^3−^ in the ClpC1-NTD–RUF-I complex. The functional significance of this finding remains uncertain.

#### 3.2.2. ClpC1-NTD–ECU, ClpC1-NTD-L92S/L96P and ClpC1-NTD–RUF-I: domain movement   

Alignment of the ClpC1-NTD C^α^ atoms from different structures resulted in subtle movements from the apo form to the RUF-I complex, while significant changes were seen from 3wdb to 6pbs, 6pba, 6pbq and 6ucr (Fig. 3 ▸ *c*). The largest movements were at both termini; the *y* axis has been truncated in Fig. 3 ▸(*c*). Met1 differed by up to 12 Å. In addition, helices 4–5 and 8–9 were shifted by about 3–4 Å owing to a pivot point in the 70s loop. It was observed that one conformation is captured in the apo form and the RUF-I and CYMA complexes, with a second conformation being captured when ECU was bound, in solution or in the ECU-resistant double mutant ClpC1-NTD-L92S/L96P. This domain movement is on the side opposite to the point mutations and the peptide-binding sites (Fig. 3 ▸).

Apart from the protein termini, the 70s loop is the most flexible portion of the NTD structures. The region consistently has higher *B* factors and was more challenging to refine, with unusual orientations of residues. This may reflect the true nature of the protein itself; the mobility of this loop may allow the domain movements that are observed between structures.

#### 3.2.3. ClpC1-NTD–ECU, ClpC1-NTD-L92S/L96P and ClpC1-NTD–RUF-I: N-terminus   

The N-terminus underwent a significant conformational change upon ECU binding. In the ECU complex the N-terminus is extended and the peptide tail swings around it, making significant contacts. The N-terminus is altered in the apo form owing to the presence of the His tag (Vasudevan *et al.*, 2013 ▸), but is found to be helical when complexed with RUF-I and CYMA. This conformation is retained when Met1 is covalently bound to CYMA or RUF-I (Wolf *et al.*, 2019 ▸) as well as in 6pbq, and is in a similar orientation in 6ucr and 6pba.

The N-terminal amine of Met1 forms a hydrogen bond (2.8 Å) to ECU at the carbonyl of Val2 (Fig. 2 ▸ *e*) that is present in the 12 ClpC1 molecules in the asymmetric unit. This significant bond is probably critical for the high affinity of ECU for ClpC1. The deletion of Val1, the substitution of Ile3 by Val and the absence of an *N*-methyl group (Val2) in OMS-A (Fig. 5 and Supplementary Fig. S2) resulted in slightly reduced affinity, as described below (Kim *et al.*, 2017 ▸). Interestingly, OMS-B, which differs by an additional methyl group at the end of its tail, showed a tenfold reduced binding. These subtle structural differences cause a major reduction in binding and indicate that N-terminal interactions are very important for the binding of ECU to ClpC1 (Supplementary Fig. S1 and Table S2).

### 3.3. Oligomeric states of ClpC1-NTD–ligand complexes   

The oligomeric aggregates found in the crystal environment of ClpC1-NTD in complex with two different cyclopeptides were examined to assess the possible relevance of their mode of action in modulating the proteolytic machinery.

**Figure 4:**
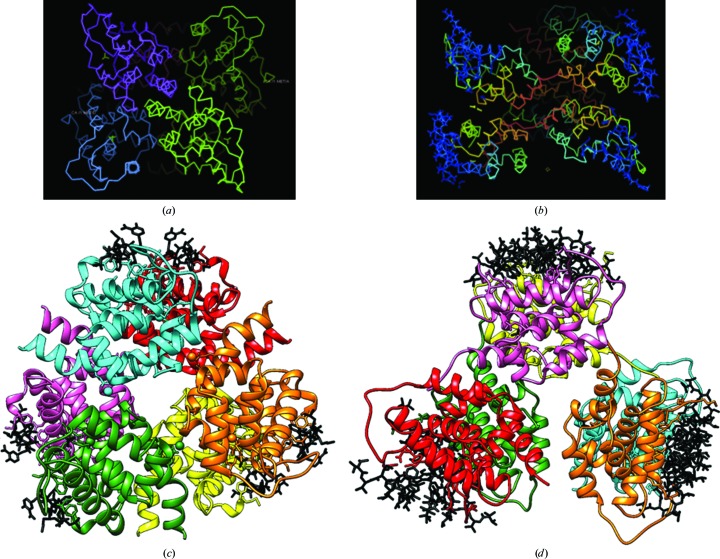
Crystal packing of the ClpC1-NTD–RUF-I and ClpC1-NTD–ECU complexes. (*a*) The C^α^ trace of the protein in ClpC1-NTD–RUF-I depicts a close association of subunits. (*b*) The ClpC1-NTD–ECU complex has much more loosly associated subunits with no stable quaternary structure predicted. Both structures are a dimer of trimers or 32 symmetry. While the RUF-I complex has a staggered orientation (*c*), the ECU complex is eclipsed (*d*).

Analysis of the cubic crystal (space group *P*4_1_32) of the ClpC1-NTD–RUF-I complex using *PISA* suggested that a hexameric oligomer, Δ*G*°_diss_ = 32.1 kcal mol^−1^, with 32 symmetry (Figs. 4 ▸ *a* and 4 ▸ *c*) could be stable in solution, with interacting surfaces ranging from approximately 100 to 640 Å^2^ and a total buried surface of 14 460 Å^2^, which is 37% of the total available surface area. This hexameric aggregate is compact, and is rather rigid based on its external appearance and low overall *B* factor derived from the Wilson plot (18 Å^2^), as expected from the high-resolution diffraction of the crystals (Figs. 4 ▸ *a* and 4 ▸ *c*). The presence of the covalent bond between the SD atom of Met1 and the open epoxide introduces additional rigidity to the complex. The complex can be described as consisting of two layers of staggered trimers, although based on the relative surface areas it is possible that individual dimers are probably the nucleating unit, driven by twofold interactions along the C-terminal helices (Figs. 4 ▸ *a* and 4 ▸ *c*).

The structure of the ClpC1-NTD–ECU complex is unique in that it consists of ClpC1-NTD and two ECU molecules related by an intramolecular dyad. In the packing of the long and extended asymmetric unit, clusters of three dimers arrange themselves in a looser hexameric arrangement. This noncrystallographic arrangement was first noticed by looking at the self-rotation function (Fig. 1 ▸) and can be loosely described as a weak clustering of two ‘S-shaped’ disks facing each other on the concave side (Fig. 4 ▸ *b*, top). *PISA* analysis revealed that possibly only the dimer would be stable in solution. The overall description of this aggregate could be a loose aggregate (the overall *B* factor from the Wilson plot is 60 Å^2^) of three dimers around a common threefold center inclined with respect to the crystal axis (Fig. 1 ▸). It is plausible that the presence of the two ECU molecules helps to stabilize this aggregate via the hydrophobic side/edge of the ECU cyclic scaffold.

While both of these protein structures have apparent 32 symmetry in the crystal packing, one has a tight staggered conformation (RUF-I-bound; Figs. 4 ▸ *a* and 4 ▸ *c*), while the other is eclipsed and much looser (ECU-bound; Figs. 4 ▸ *b* and 4 ▸ *d*). ClpC1-NTD–RUF-I crystallized in the presence of 2.5 *M* NaCl, which could increase stability and result in the strong hexameric association. None of the other ClpC1-NTD structures, either apo or complexed with CYMA, were predicted to form quaternary associations by *PISA*, and all of the structures crystallized under much lower salt conditions, similar to the ECU complex.

### 3.4. SPR binding studies   

#### 3.4.1. One-site versus two-site binding of ECU   

**Figure 5:**
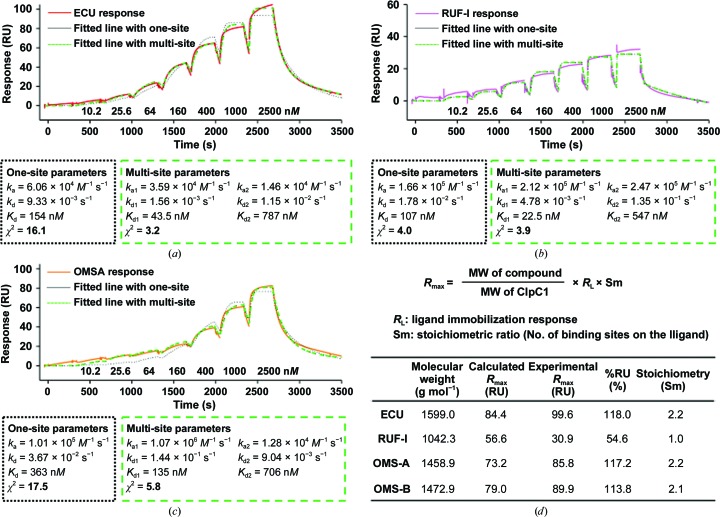
Cyclopeptide-binding analysis by SPR. The experimental binding sensorgrams were compared with two kinetic binding models, the 1:1 Langmuir and the heterogeneous ligand (multi-site) binding models, for ECU (*a*), RUF-I (*b*) and OMS-A (*c*) binding to ClpC1-FL. (*d*) The stoichiometry was calculated based on SPR responses.

As the structure of the ECU complex contained two molecules of ECU, SPR binding data were fitted to determine the model that was in best agreement with the data. Fig. 5 ▸(*a*) shows the ECU binding response to ClpC1-FL in solid red, and two fitted lines constructed using the 1:1 Langmuir (one-site) and heterogeneous ligand (multi-site) models are shown as dotted (black) and dashed (green) lines, respectively. The goodness of fit can be compared based on χ^2^ values, with smaller χ^2^ values representing a better fit. The χ^2^ values from one-site and multi-site fitting for ECU were 16.1 and 3.2, respectively, indicating that the multi-site model agrees better with the obtained sensorgram data than the one-site model. The dashed line (green) fits the ECU sensorgram (red) better than the dotted black line in Fig. 5 ▸(*a*), reflecting a fivefold smaller χ^2^ value of the multi-site fit. The same analysis was applied to RUF-I and OMS-A (a slightly shorter ECU analog) for comparison. The two fitted lines for RUF-I were almost identical, resulting in similar χ^2^ values (Fig. 5 ▸ *b*). In contrast, the OMS-A sensorgram was similar to that of ECU and fitted better using a multi-site binding model (Fig. 5 ▸ *c*), with threefold better χ^2^ values.

The SPR binding response is proportional to the mass on the sensor surface when molecules bind to the immobilized binding partners. In previously unpublished experiments, the *R*_max_ values were always higher for ECU than RUF-I, even after taking into account the molecular-weight differences. The stoichiometry was calculated for these four compounds based on binding response. The theoretical *R*_max_ values can be calculated based on the molecular weights of the compounds and the immobilized protein and the immobilization level of ClpC1 on each sensor surface; the equation used to calculate *R*_max_ is shown in Fig. 5 ▸(*d*). According to the published X-ray structure of RUF-I in complex with ClpC1-NTD (Wolf *et al.*, 2019 ▸), one RUF-I molecule binds to ClpC1-NTD. The stoichiometric ratios of both ECU and OMS-A to ClpC1 were determined to be 2.2, suggesting that two ECU or two OMS-A molecules bind to a single ClpC1-NTD. The same binding mode was seen for OMS-B. This is in agreement with the newly solved ECU complex structure.

The multi-site analysis of the binding of ECU and OMS-A also provides support for the differences in the binding of ECU and OMS-A at the two different sites. The *K*_d1_ values for ECU and OMS-A are 43.5 and 135 n*M*, respectively, compared with *K*_d2_ values for the second site of 787 and 706 n*M*, respectively. This would suggest that site 1 of ECU has a higher affinity than site 2, and that the affinity for either ECU or OMS-A is comparable in site 2. The shorter ECU tail in OMS-A could explain the lower affinity for the latter, given the importance of the N-terminal contacts in the ClpC1-NTD–ECU complex. Once site 1 is occupied, the difference in the affinities for the second site in either ECU or OMS-A is not significant. Furthermore, the *K*_d1_/*K*_d2_ values for ECU differ by about 18-fold, a significant difference compared with the corresponding *K*_d_ ratios of OMS-A, which only differ by fivefold and can be explained by the lower affinity of site 1 for OMS-A. OMS-B binding to ClpC1-FL was much weaker with *K*_d1_ and *K*_d2_ values of 962 and 1740 n*M*.

#### 3.4.2. ECU site mutants   

**Figure 6:**
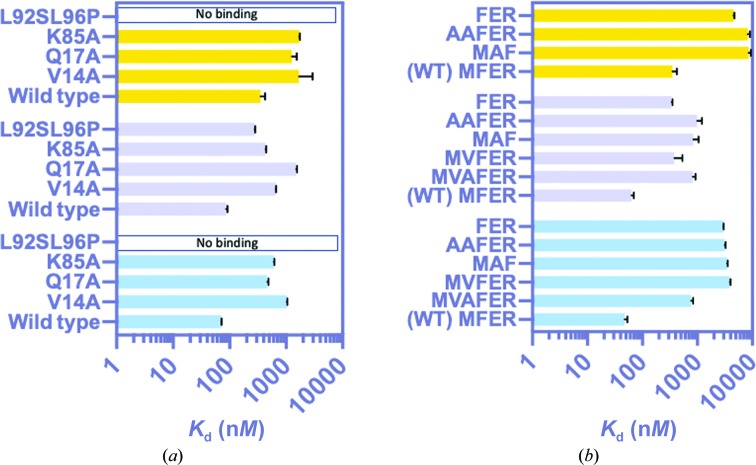
SPR binding of ClpC1 mutants to macrocyclic peptides. (*a*) ClpC1-FL with various sites mutated was immobilized, while the cyclopeptide (ECU in blue, RUF-I in lilac and OMS-A in yellow) was passed over the surface. Averages of three titrations are shown with standard deviations in black. (*b*) ClpC1-NTD with mutations at the N-terminus was also titrated with the cyclopeptides. Wild-type (WT) results are plotted for reference.

Recently, ECU binding has been shown to be disrupted by the single mutations L92S, L92F and L96P (Wolf *et al.*, 2019 ▸), with a 70-fold to 244-fold reduction in binding to ECU but with negligible effects on RUF-I binding (an 0.7-fold to twofold reduction). The double-mutant protein ClpC1-NTD-L92S/L96P abolished binding to ECU and OMS-A (Fig. 6 ▸ *a* and Supplementary Table S4), while the RUF-I binding affinity was reduced by threefold. After the discovery of the second ECU binding site, additional sites were mutated to indicate the essentiality of these residues for site 2 binding and to confirm that this binding site was not an artifact of crystallization.

In the X-ray structure, His77 and Gln17 were found to hydrogen-bond to ClpC1-NTD at ECU site 2. Val14 is 3.9 Å away from the phenyl ring of ECU2, whereas Lys85 is the residue closest to the indole (3.7 Å; ECU2 CDC to Lys85 CD). The V14A and K85A mutations had a reduced binding affinity for ECU (by 14-fold and ninefold, respectively) and had a slightly more modest effect on RUF-I binding (an eightfold and fivefold reduction, respectively) (Fig. 6 ▸ *a*, Supplementary Table S4). Interestingly, the Q17A mutation reduced ECU binding by sevenfold and RUF-I binding by 17-fold. OMS-A binding was reduced by fourfold to sevenfold for the three ECU site 2 mutants.

The N-terminus had a critical impact on the binding of both ECU and RUF-I (Fig. 6 ▸ *b* and Supplementary Table S5). While forming a covalent bond with Met1 (PDB entry 6cn8), RUF-I binding is less affected than ECU binding. OMS-A and OMS-B are structurally similar to ECU but reduce binding by twofold and tenfold, respectively. OMS-A also has significant antimicrobial activity against *Mtb* (Kim *et al.*, 2019 ▸), with a minimum inhibitory concentration (MIC_90_) of 270 ± 37 n*M*; the MIC_90_ of ECU was tested in parallel, with a value of 110 ± 13 n*M*. The MIC_90_ values for OMS-A showed a pattern consistent with ECU for *Mtb* strains resistant to RUF-I and ECU (unpublished data). Engineered mutants were purified to investigate the importance of the N-terminal residue. Inserting Val before Phe2 reduced ECU binding the most (82-fold), while insertion of Ala at this position decreased binding 72-fold. Deleting Met1 reduced the ECU binding affinity by 62-fold, and a Met1Ala mutation and Ala insertion caused a 68-fold reduction. For RUF-I, deletion of Met1 had the smallest effect of a fivefold reduction in binding, whereas a Val insertion only reduced the affinity by sixfold. However, the Ala and ValAla insertions and the Met1Ala substitution and Ala insertion had the greatest reductions in affinity at 13-fold to 16-fold. In summary, all N-terminal mutations had a greater effect on ECU binding, except for the ValAla insertion, which produced about the same effect. The effect of these mutations on OMS-A binding was intermediate, with a 25-fold to 46-fold reduction in binding.

## 4. Discussion   

### 4.1. Implications of the SPR results for the *in vivo* mode of action   

While previous results have indicated that ECU and RUF-I have similar binding affinities for ClpC1, their rates of association/dissociation exhibited significant differences in the presented SPR measurements. RUF-I is known to form a covalent adduct with the N-terminus, creating a stable connection; however, this reaction was too slow to be observed by SPR experiments (Wolf *et al.*, 2019 ▸). Additional prior observations concluded that RUF-I cannot bind in the presence of ECU, presumably owing to the much slower dissociation of ECU. Accordingly, the two peptides must share a portion of the target binding pocket. The X-ray structures of both complexes that are now available enhance the understanding of the two different modes of binding to the same area of the target and allow further insights into their possible modes of action.

In addition, the SPR data on the mutants and the multi-site binding of ECU to ClpC1-FL provided further strong evidence for the unusual stoichiometry of the ClpC1-NTD–ECU complex (1:2) in solution. The unique binding that has been demonstrated reflects binding of ECU to the complete ClpC1 protein and is not an artifact induced by the crystal packing of the smaller NTD domain. Moreover, the ECU complex structure identified the N-terminus as being critical for binding. ECU has a greater than tenfold higher affinity for ClpC1 than OMS-B, which differs by one less amino acid, by the replacement of Ile3 by Val and by lacking the *N*-methyl substituent in Val2 of ECU. These seemingly small changes significantly reduced the binding affinity and *in vivo* activity.

Proline residues are known to introduce kinks in helical stretches by disrupting the typical hydrogen bonding between the NH of residue *i* and the C=O of residue *i* + 4 along the helical path (Reiersen & Rees, 2001 ▸). However, in ClpC1 the L96P mutant confers ECU resistance, as do the L92S and L92F mutations. As shown in Fig. 3 ▸(*b*), it was observed that the double mutation L92S/L96P introduced a kink in helix 6 and replaces a hydrophobic residue (Leu) with a more polar Ser. This double mutation results in the alteration of an important interaction with the side chain of Arg4 and therefore is the most probable cause of resistance to ECU. Proline mutations have been found to stabilize a protein and impede the ability to develop resistance (Loo & Clarke, 1993 ▸) or to help confer resistance in other cases (Yin *et al.*, 2007 ▸).

### 4.2. Implications for the mode of action of therapeutic agents targeting the ClpC1–ClpP1–ClpP2 system   

The complex structures of the two different types of cyclopeptides with the N-terminal domain of their putative target, ClpC1-NTD, revealed two different strategies to interact with this smaller fragment, as well as two different types of alterations of the relative orientation of the ClpC1 element in the proteolytic machinery of the proteolysis unit. The entire assembly consists of two heptameric pieces of ClpP1 and ClpP2 forming an overall tetradecameric cylindrical-shaped barrel, upon which the ClpC1 unit binds (Li *et al.*, 2016 ▸) (PDB entries 5dzk and 5e0s; Gatsogiannis *et al.*, 2019 ▸). The presence of these two different classes of active agents could alter the position and/or orientation of the ‘modulator’ unit (ClpC1) with respect to both the proteolytic machinery (ClpP1–ClpP2) and possibly the coupling between the ATPase activity, residing in domains D1 and D2 of full-length ClpC1, and the proteolytic activity located in the ClpP1–ClpP2 barrel-like structure.

**Figure 7:**
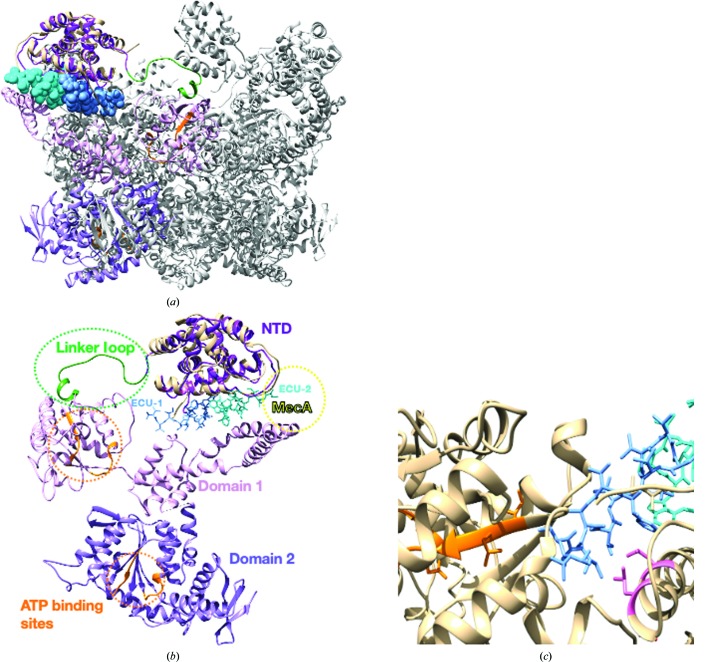
Quaternary-structural insights from comparison of *B. subtilis* ClpC–MecA and *Mtb*ClpC1-NTD–ECU structures. (*a*) The ClpC hexamer from *B. subtilis* (PDB entry 3j3s) is shown in gray; one monomer is in color. *Mtb*ClpC1-NTD–ECU can be seen in beige with ECU molecules as spheres at the NTD–D1 interface. (*b*) A single monomer from PDB entry 3j3s is shown with each domain in a different shade of purple. ClpC1-NTD–ECU is in beige with ECU site 1 in cornflower blue and ECU site 2 in cyan. The binding site of the bound MecA is shown in yellow (MecA was removed for simplicity). A longer linker loop (green) connects the NTD to domain D1. The ATP-binding regions (Walker A and B) in domains D1 and D2 are colored orange. (*c*) ECU site 1 (cornflower blue) occupies the space at the beginning of the Walker A region (orange). The ECU-resistance sites Leu92 and Leu96 are in pink; Leu96 is in the foreground. The atoms of ECU1 overlap with the D1 domain.

Based on the cryo-EM structure of *B. subtilis* ClpC (PDB entry 3j3s; Fig. 7 ▸ *a*), the peptide-binding site is at the interface of the NTD and domain D1 (Liu *et al.*, 2013 ▸). However, a 20-residue loop links these two domains and is probably quite mobile (Fig. 7 ▸ *b*). This linker loop is oriented towards the center of the pore, which may allow the NTDs to swing in towards each other, ‘closing’ the pore. At this point it remains unknown whether the NTD is typically found in the captured orientation, as in this structure MecA binds at the interface of the NTD and domain D1, possibly locking contact between the two domains. Superposing our ClpC1-NTD–ECU structure onto PDB entry 3j3s, ECU occupied the space at the beginning of the β-strand of the Walker B region of the ATP-binding site in domain D1 (Fig. 7 ▸ *c*). It has previously been shown that ECU enhances the ATPase activity of ClpC1 by at least twofold, whereas RUF-I has no significant effect (Choules *et al.*, 2019 ▸). ECU binding may open the space between the NTD and D1 and allow ATP to have greater access to its binding pocket.

The smaller heptapeptides, CYMA and RUF-I, form a bridge over a hydrophobic ridge dominated by the aligned phenyl rings of Phe2 and Phe80. This bridge is rigid, and the binding of the main scaffold of the two cyclopeptides is near-identical, although RUF-I has a six-membered ring which provides additional rigidity. The atomic footprint of the two heptapeptides on the surface of ClpC1-NTD is essentially the same (∼580 Å^2^). Intriguingly, the two heptapeptides contain an epoxide extension from the distinct ‘indole-like’ ring that appears to be open and linked via a covalent adduct to the SD atom of Met1 in both complex crystal structures. The significance of this observation for the *in vitro* and *in vivo* activity of these compounds is still uncertain. Extending out of this β-strand scaffold, both heptapeptides contain various side chains that typically alternate between Val and Leu and the larger aromatic side chains nitro-Tyr or β-hydroxy-Phe. The effect of these protrusions on the relative position and orientation of the following D1 and D2 domains forming the full ClpC1 is unknown, although it is reasonable to assume that they regulate the entry(ies) to the proteolytic chamber of the entire assembly.

In contrast, ECU is a tridecamer depsipeptide with a larger scaffold and atomic footprint (600 Å^2^). ECU also has an extended tail of three amino acids that protrudes from the structure of the complex and plays a significant role in binding the amino-terminus of ClpC1-NTD. Moreover, the unique mode of ClpC1-NTD–ECU binding results in what could be an extended and massive disturbance of the relative orientation and position of the D1 and D2 domains of ClpC1. Although sharing common features related to the inter-domain dyad of the helical structure of ClpC1-NTD, the two ECU-binding sites are significantly different. The most notable difference is related to the way that the N-terminus of ClpC1-NTD (Met1-Phe2-Glu3-Arg4) adopts an unusually extended conformation that is induced by the formation of hydrogen-bond interactions with the more polar side of the ECU molecule (Fig. 2 ▸ *e* and Supplementary Fig. S1). The result is that the N-terminus of ClpC1-NTD undergoes a distinct polar interaction with a C=O group in the extended tail of ECU. This critical interaction is not present in the ECU analogs related to OMS-A, making it a weaker binder, as observed in the SPR binding studies.

The other side of the ECU molecule contains four *N*-methyl groups, eliminating the possibility of hydrogen bonds, but creating a hydrophobic ‘strip of contact’ (∼240 Å^2^) for the adjacent ECU molecule across the intermolecular dyad (Figs. 2 ▸ *d* and 2 ▸ *e*). This unique binding relationship would more likely make the binding of the two ECU molecules cooperative, as suggested by ATPase activity measurements (Gao *et al.*, 2015 ▸). It is noteworthy that the two different classes of molecules seem to be active by trapping or sequestering the N-terminus of ClpC1 and possibly preventing its interaction with other structural elements in ClpC1 itself or in the more extended ClpC1–ClpP1–ClpP2 complex. More extensive and detailed experimental studies using high-resolution SAXS and cryo-EM with the intact particles of the ClpC1–ClpP1–ClpP2 unit in the presence of RUF-I, CYMA, OMS-A and ECU are planned in order to understand the conformational changes induced by these potent agents and the functional significance of the N-terminal residues of ClpC1.

*Note added in proof*. Due to a hidden minor error, the stereochemistry of the C_β_ carbons for residues Ile3 and Thr5 of the ecumicin structures in the original 6pbs deposition has been encoded incorrectly. This error does not affect in any significant way the structural findings derived from this work. A revised 6pbs deposition with the correct configurations of Ile3 and Thr5 is currently in progress.

## Supplementary Material

PDB reference: ClpC1-NTD, 6pba

PDB reference: 6pbq

PDB reference: complex with ecumicin, 6pbs

PDB reference: L92S/L96P double mutant, 6ucr

Supplementary Tables and Figures. DOI: 10.1107/S2059798320004027/dw5208sup1.pdf

## Figures and Tables

**Table 1:** Data collection and refinement statistics Values in parentheses are for the highest resolution shell.

	6pbs	6ucr	6pba	6pbq
Wavelength (Å)	0.97856	0.97872	0.97856	0.97856
Resolution (Å)	19.49–2.50 (2.59–2.50)	33.30–2.29 (2.37–2.29)	31.74–1.90 (1.97–1.90)	30.38–1.60 (1.66–1.60)
Space group	*P*2_1_	*P*2_1_2_1_2_1_	*C*2	*P*2_1_
*a*, *b*, *c* (Å)	80.063, 130.342, 112.562	38.137, 63.35, 68.305	44.444, 59.235, 58.833	33.663, 63.403, 36.752
β (°)	90.07		97.472	115.527
Total reflections	371245	96494	51401	62231
Unique reflections	77925 (6574)	7562 (744)	11895 (1139)	17301 (1798)
Multiplicity	4.09 (2.25)	12.7 (14.0)	4.3 (3.9)	3.6 (3.7)
Completeness (%)	97.87 (83.62)	96.13 (96.88)	98.70 (93.69)	93.88 (98.74)
Mean *I*/σ(*I*)	14.54 (1.81)	56.149 (27.159)	41.53 (4.05)	21.00 (8.73)
Wilson *B* factor (Å^2^)	55.18	19.02	31.64	12.49
*R*_meas_	0.071 (0.612)	0.100 (0.253)	0.063 (0.492)	0.062 (0.117)
CC_1/2_	0.997 (0.707)	0.995 (0.990)	0.983 (0.890)	0.991 (0.980)
Reflections used in refinement	77887 (6573)	7560 (744)	11869 (1128)	17299 (1798)
Reflections used for *R*_free_	3796 (339)	357 (40)	583 (60)	818 (106)
*R*_work_	0.188 (0.355)	0.199 (0.196)	0.202 (0.328)	0.178 (0.161)
*R*_free_	0.267 (0.414)	0.234 (0.321)	0.212 (0.318)	0.212 (0.214)
No. of non-H atoms				
Total	17311	1299	1258	1358
Macromolecules	15345	1193	1177	1180
Ligands	1076	4	—	20
Solvent	890	102	81	158
Protein residues	1754	154	152	152
R.m.s.d. from ideal				
Bond lengths (Å)	0.024	0.014	0.015	0.013
Angles (°)	2.26	1.77	1.85	1.91
Ramachandran statistics				
Favored (%)	94.10	98.68	96.67	98.67
Allowed (%)	4.80	1.32	3.33	1.33
Outliers (%)	1.10	0.00	0.00	0.00
Clashscore	10.54	4.55	2.50	10.22
