# Supplementary material for: Structure of the N-terminal domain of ClpC1 in complex with the antituberculosis natural product ecumicin reveals unique binding interactions
Source: Acta Crystallogr D Struct Biol. 2020 Apr 23;76(Pt 5):458–71. doi: 10.1107/S2059798320004027 (PMC7193532; doi:10.1107/S2059798320004027)
Supplement: Supplementary file 1 [file d-76-00458-sup1.pdf]

STRUCTURAL BIOLOGY

Volume 76 (2020)

Supporting information for article:

Structure of the N-terminal domain of ClpC1 in complex with the antituberculosis natural product ecumicin reveals unique binding interactions

Nina M. Wolf, Hyun Lee, Daniel Zagal, Joo-Won Nam, Dong-Chan Oh, Hanki Lee, Joo-Won Suh, Guido F. Pauli, Sanghyun Cho and Celerino Abad-Zapatero

Table S1 RMS and average deviations of the C_α carbons of the 12 ClpC1-NTD molecules within the asymmetric unit of 6PBS for residues 1-144, aligned to chain W (reference).

A total of 66 possible unique combinations are possible. The superposition of chain W against the eleven others was considered to be a reasonable sampling for possible variations in RMS and average deviations.

Chain	Chain W RMSD (Å)	Chain W Average Deviations (Å)
A	0.46	0.27
I	0.29	0.24
C	0.54	0.36
Y	0.44	0.28
e	0.48	0.30
B	0.35	0.25
G	0.30	0.25
K	0.47	0.35
M	0.24	0.19
O	0.48	0.31
T	0.59	0.33
Mean	0.42 ± 0.11	0.28 ± 0.15

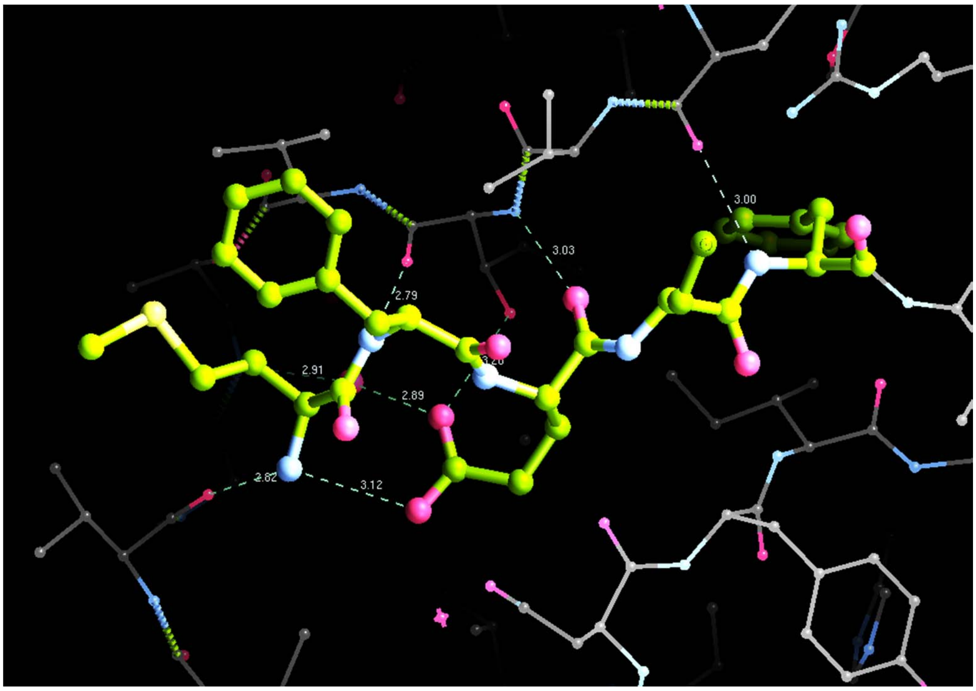

Figure S1 N-terminal (residues 1-5: Met-Phe-Glu-Arg-Phe, ball and sticks) hydrogen bond interactions between ClpC1-NTD and ECU site 1 (thin bonds) (see Table below).

Table S2 Distances between the N-terminus (a.a. 1-5) of the ClpC1-NTD-ECU complex (chain W) and the nearby atoms (range 2.7-3.4 Å) of the ECU site 1 (chain N), illustrating the hydrogen bonding network between protein and ligand.

ClpC1-NTD-Atom 1	ECU-Atom 2	Distance (Å)
Met1-N	Val2-CO	2.82
Phe2-N	12(β-hydroxy-Phe)-OXT	2.79
Glu3-CO	12(β-hydroxy-Phe)-N	3.03
Glu3-OE1-Met1-N**	-	3.12
Glu3-OE2	12(β-hydroxy-Phe)-OB	3.20
Glu3-OE2-W102*	Thr4-N	2.91
Phe5-N	Trp10-ODJ	3.0

*Water mediated hydrogen bond to ECU.

**Intramolecular hydrogen bond in ClpC1-NTD.

Table S3 RMS (blue) and average deviations (yellow) of the C_α carbons of ClpC1-NTD (residues 1-145) from various structures.

The most significant movement was seen at the N-terminus when ECU is bound. Therefore, some calculations were repeated omitting residues 1-3 (N-terminus) from the superposition, values in parenthesis.

	3wdb	3wdc	6cn8	L92SL96P	6PBA	6PBQ	6PBS-chain W
3wdb	-	0.81	0.83	2.06	2.00	2.07	2.45
3wdc	0.57	-	0.73	1.69	1.58	1.64	1.98
6cn8	0.49	0.61	-	1.91	0.73	1.98	2.18
L92SL96P	1.81	1.52	1.74	-	1.69	0.93	1.12 (0.71)
6PBA	1.65	1.39	0.61	1.52	-	0.72	1.38 (0.43)
6PBQ	1.77	1.44	1.68	0.56	0.46	-	1.36 (0.68)
6PBS-chain W	1.94	1.61	1.82	0.61 (0.52)	0.62 (0.37)	0.69 (0.44)	-

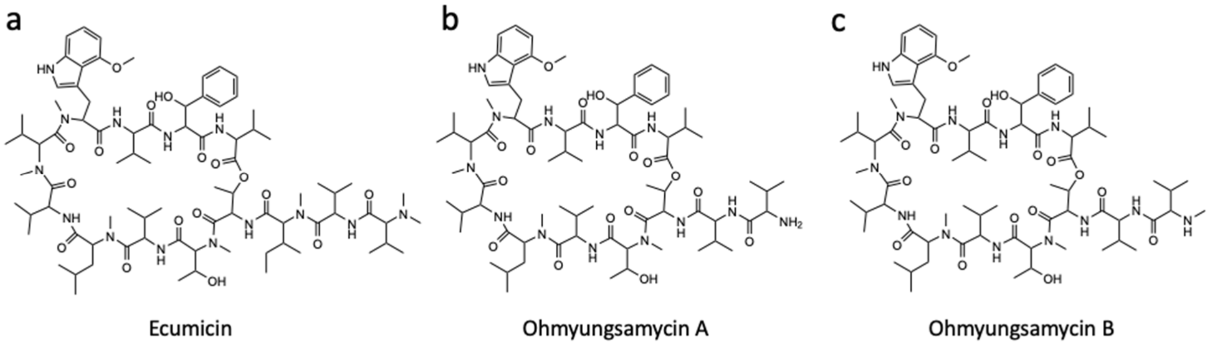

Figure S2 Two-dimensional drawings of the structurally related ecumicin (a), ohmyungsamycin A (b) and ohmyungsamycin B (c).

Table S4 Kinetic binding of macrocyclic peptides to ClpC1-FL with various mutations along the amino acid sequence were fit to a 1:1 Langmuir binding equation.

No binding (NB) was detected for ECU and OMS-A binding with the double mutant protein.

	K_D (nM)			k_a ($M^{-1}s^{-1}$)			k_d (s^{-1})		
Mutation	ECU	OMS-A	RUF-I	ECU	OMS-A	RUF-I	ECU	OMS-A	RUF-I
V14A	1030 ± 22	1690± 1257	654± 9	$1.42x10^4$ ± $0.93x10^4$	$8.96x10^3$ ± $1.05x10^3$	$1.34x10^5$ ± $0.21x10^5$	$1.46x10^{-2}$ ± $0.12x10^{-2}$	$2.16x10^{-2}$ ± $0.29x10^{-2}$	$8.77x10^{-2}$ ± $0.02x10^{-2}$
Q17A	465 ± 24	1260± 280	1500 ± 62	$9.67x10^3$ ± $0.405x10^3$	$1.79x10^4$ ± $1.29x10^4$	$7.77x10^4$ ± $0.47x10^4$	$4.50x10^{-3}$ ± $0.38x10^{-3}$	$2.03x10^{-2}$ ± $1.35x10^{-2}$	$1.17x10^{-1}$ ± $0.12x10^{-1}$
K85A	607 ± 14	1720 ± 36	433± 10	$1.61x10^4$ ± $0.16x10^4$	$8.44x10^3$ ± $2.74x10^3$	$8.62x10^4$ ± $0.91x10^4$	$9.69x10^{-3}$ ± $1.16x10^{-3}$	$1.45x10^{-2}$ ± $0.45x10^{-2}$	$3.73x10^{-2}$ ± $0.31x10^{-2}$
L92SL96P	NB	NB	276± 9	-	-	$2.81x10^5$ ± $1.18x10^5$	-	-	$7.73x10^{-2}$ ± $3.07x10^{-2}$

Table S5 Kinetic binding of macrocyclic peptides to ClpC1-NTD with various mutations (left most column) in the N-terminus were fit to a 1:1 Langmuir binding equation.

OMS-A was not tested (NT) with MVFER and MVAFER mutant ClpC1-NTD.

	K_D (nM)			k_a ($M^{-1}s^{-1}$)			k_d (s^{-1})		
Mutation	ECU	OMS-A	RUF	ECU	OMS-A	RUF	ECU	OMS-A	RUF
MAFER	3500 ± 90	8520± 883	848 ± 201	$2.14x10^3$ ± $0.166x10^3$	$2.83x10^3$ ± $0.228x10^3$	$3.90x10^4$ ± 1.15 $x10^4$	$7.47x10^{-3}$ ± $0.5x10^{-3}$	$2.40x10^{-2}$ ± $0.08x10^{-2}$	$3.42x10^{-2}$ ± $1.76x10^{-2}$
MVFER	3930 ± 72	NT	383± 150	$1.10x10^3$ ± $0.040x10^3$	-	$2.95x10^4$ ± $0.53x10^4$	$4.32x10^{-3}$ ± $0.2x10^{-3}$	-	$1.08x10^{-2}$ ± $0.20x10^{-2}$
MVAFER	771± 67	NT	827± 102	$1.50x10^4$ ± $0.42x10^4$	-	$1.75x10^4$ ± $0.39x10^4$	$1.19x10^{-2}$ ± $0.17x10^{-2}$	-	$1.71x10^{-2}$ ± $0.08x10^{-2}$
FER	2960 ± 25	4520± 206	342 ± 10	$1.22x10^3$ ± $0.023x10^3$	$1.36x10^3$ ± $0.066x10^3$	$1.40x10^5$ ± $0.92x10^5$	$3.60x10^{-3}$ ± $0.06x10^{-3}$	$4.40x10^{-3}$ ± $3.31x10^{-3}$	$3.36x10^{-2}$ ± $2.50x10^{-2}$
AAFER	3150 ± 107	8290 ± 737	997 ± 219	$1.44x10^3$ ± $0.037x10^3$	$1.07x10^3$ ± $1.33x10^3$	$8.35x10^4$ ± $1.68x10^4$	$4.55x10^{-3}$ ± $0.3x10^{-3}$	$8.92x10^{-3}$ ± $1.65x10^{-3}$	$8.49x10^{-2}$ ± $3.02x10^{-2}$
